# Genome Sequence of a *Burkholderia pseudomallei* Clinical Isolate from a Patient with Community-Acquired Pneumonia and Septicemia

**DOI:** 10.1128/genomeA.00915-15

**Published:** 2015-08-20

**Authors:** Chiranjay Mukhopadhyay, K. E. Vandana, T. A. K. Chaitanya, Tushar Shaw, H. Vinod Bhat, Sanjiban Chakrabarty, Bobby Paul, Sandeep Mallya, T. S. Murali, Kapaettu Satyamoorthy

**Affiliations:** a Department of Microbiology, Kasturba Medical College, Manipal University, Manipal, Karnataka, India; b Department of Community Medicine, Kasturba Medical College, Manipal University, Manipal, Karnataka, India; c Department of Biotechnology, School of Life Sciences, Manipal University, Manipal, Karnataka, India

## Abstract

Here, we report the draft genome sequence of *Burkholderia pseudomallei* CM_Manipal, the causative agent of melioidosis isolated from a diabetic patient in Manipal, southern India. The draft genome consists of 107 contigs and is 7,209,157 bp long. A total of 5,600 coding sequences (CDSs), 60 tRNAs, 12 rRNAs, and one noncoding RNA (ncRNA) were predicted from this assembly.

## GENOME ANNOUNCEMENT

*Burkholderia pseudomallei* is an aerobic saprophytic Gram-negative bacillus and is the causative agent of melioidosis. This disease is endemic in the top end of the northern territory of Australia, northeastern Thailand, Malaysia, Singapore, Vietnam, Cambodia, and Laos (1). There are increasing reports of cases beyond the regions in which it is endemic, including from the Indian subcontinent, southern China, Hong Kong, Taiwan, various Pacific and Indian Ocean islands, and some parts of Africa and America (2). It primarily affects people who are in regular contact with soil and water due to agricultural activities, mostly during rainy seasons, through percutaneous inoculation, inhalation, or ingestion (3). Melioidosis may evolve as localized or systemic infections in the form of pneumonia, bacteremia, bone and joint infections, skin infections, deep organ abscesses, genitourinary tract infections, and, rarely, neurological infections. The usual comorbidities are long-standing diabetes mellitus, heavy alcohol use, chronic pulmonary and renal diseases, steroid therapy, and cancer. The mortality rate of melioidosis ranges from 14% to 40% (4, 5). The Indian subcontinent is suggested to be endemic (6), although there is paucity of data in the domain of molecular epidemiology from the country.

Here, we report the complete genome sequence of *B. pseudomallei* strain CM_Manipal, isolated from a 55-year-old diabetic male patient admitted to Kasturba Medical College Hospital, Manipal with chief complaints of fever and chills for 3 days. On the third day of admission, the patient developed seizures and was intubated. His chest x-ray showed bilateral diffuse alveolar opacities, and his total leukocyte count was 28,000/mm_3_. Endotracheal aspirate (ETA) was cultured by both conventional and enrichment techniques. The bacterial isolate was identified as *B. pseudomallei* using standard biochemical tests and later confirmed using an automated microbiological identification system (Vitek 2 Compact; BioMérieux, Eliot, France) and a monoclonal antibody-based latex agglutination test (Mahidol University, Bangkok, Thailand). Later, the blood culture also showed the presence of *B. pseudomallei*. The strain was susceptible to ceftazidime, trimethoprim-sulfamethoxazole, doxycycline, amoxicillin-clavulanic acid, and meropenem and was resistant to gentamicin.

Genomic DNA from *B. pseudomallei* was isolated using a QIAamp DNA minikit (Qiagen, Germany), and whole-genome sequencing was performed using an Ion Torrent personal genome machine (Life Technologies, CA) per the manufacturer’s guidelines. Preliminary *de novo* assembly of raw short reads (2,902,789) was performed using the MIRA4.0.2 assembler (7). Subsequently, the contigs were merged based on overlapping regions, and gaps were filled with unmapped reads in *de novo* assembly using a Bowtie 2 aligner (8) with *Burkholderia pseudomallei* NAU20B-16 as the reference genome (GenBank accession numbers CP004003.1 and CP004004.1). The draft genome consists of 107 contigs with an *N*_50_ of 135,886 bp. The largest contig was 542,166 bp long, and the shortest was 631 bp long. The size of the draft genome is 7,209,157 bp and has a G+C content of 68.06%. The functional annotation of the assembled genomes was performed by the Prokaryotic Genome Annotation Pipeline v2.10 (NCBI) for deposition with the genome database. A total of 6,273 protein coding genes predicted from this genome included 600 pseudogenes, 60 tRNAs, 12 rRNAs, and one noncoding RNA (ncRNA).

### Nucleotide sequence accession numbers.

The draft genome and annotation data for *Burkholderia pseudomallei* have been submitted to GenBank under the accession number LAUB02000001 (BioProject number PRJNA278506, BioSample number SAMN03420870).
